# Behavioral and neurophysiological effects of electrical stunning on zebrafish larvae

**DOI:** 10.1038/s41684-024-01505-0

**Published:** 2025-01-27

**Authors:** David-Samuel Burkhardt, Claire Leyden, Carina Thomas, Christian Brysch, Florian Alexander Dehmelt, Aristides B. Arrenberg

**Affiliations:** Werner Reichardt Centre for Integrative Neuroscience and Institute for Neurobiology, University of Tuebingen, Tuebingen, Germany

**Keywords:** Lab life, Ethics, Law, Cell death in the nervous system, Sensorimotor processing

## Abstract

Two methods dominate the way that zebrafish larvae are euthanized after experimental procedures: anesthetic overdose and rapid cooling. Although MS-222 is easy to apply, this anesthetic takes about a minute to act and fish show aversive reactions and interindividual differences, limiting its reliability. Rapid cooling kills larvae after several hours and is not listed as an approved method in the relevant European Union directive. Electrical stunning is a promising alternative euthanasia method for zebrafish but has not yet been fully established. Here we characterize both behavioral and neurophysiological effects of electrical stunning in 4-day-old zebrafish larvae. We identified the electric field characteristics and stimulus duration (50 V/cm alternating current for 32 s) that reliably euthanizes free-swimming larvae and agarose-embedded larvae with an easy-to-implement protocol. Behavioral analysis and calcium neurophysiology show that larvae lose consciousness and stop responding to touch and visual stimuli very quickly (<1 s). Electrically stunned larvae no longer show coordinated brain activity. Their brains instead undergo a series of concerted whole-brain calcium waves over the course of many minutes before the cessation of all brain signals. Consistent with the need to implement the 3R at all stages of animal experimentation, the rapid and reliable euthanasia achieved by electrical stunning has potential for refinement of the welfare of more than 5 million zebrafish used annually in biomedical research worldwide.

## Abstract (web summary)

This study explores the use of electrical stunning as a viable alternative to anesthetic overdose and rapid cooling for euthanizing zebrafish larvae, potentially improving the welfare of over 5 million zebrafish in biomedical research.

## Main

**Fig. 1:**
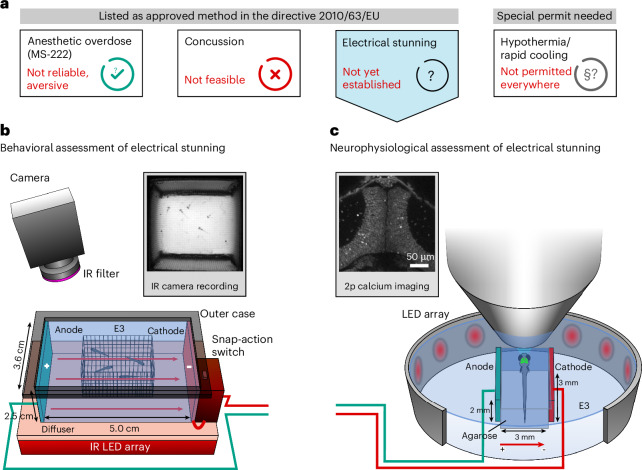
Developing a protocol for humane euthanasia via electrical stunning. **a**, An illustration of different euthanasia methods for zebrafish. **b**, The electrical stunning chamber with cuboid net container allows imaging of behavior. **c**, The brain activity of immobilized animals can be measured via 2-photon (2p) calcium imaging during visual stimulation and electrical stunning.

The zebrafish (*Danio rerio*) is one of the most common model organisms in neuroscience and biomedical research, with more than 5 million individuals used in animal experiments annually^1,2^. Because this figure excludes many animals used just for breeding instead of experiments, or animals used below the legal age limit (5 days post fertilization, or dpf), the total number is even higher. Also, it is expected that the overall number will further increase owing to the promising characteristics of this model organism and its continued spread into many areas of research^3^. Good scientific practice alone already requires well-designed and planned animal experiments^4^, but this is further reinforced by an increasing awareness of the use of animals for scientific research in society^5^ and the still unanswered question of to what extent fish perceive and suffer from pain^6–8^. Within the European Union (EU), directive 2010/63/EU states the legal euthanasia methods for fish: percussive blow to head, anesthetic overdose and electrical stunning. However, uncertainties persist concerning the application of these methods to zebrafish larvae (Fig. 1a).

First, percussive blows to the head are not practical or safe to perform because larvae are only 4 mm in size^9^. Second, overdose of the anesthetic MS-222, which in adult fish leads to death through asphyxiation and hypoxemia due to blocked gill ventilation^10^, does not reliably work on zebrafish larvae, as they rely on cutaneous gas exchange during early gill development^11,12^. At 12–14 dpf, cutaneous gas exchange is no longer sufficient to meet the minimal respiratory requirements^13^. Thus, despite the onset of a cardiac arrest when using a high concentration of MS-222 (900 mg/l), zebrafish larvae regain consciousness when transferred back to regular tank water^11^. Moreover, MS-222 triggers aversive reactions in zebrafish^14,15^. Behaviors such as piping, twitching and erratic swimming have been observed in fish when exposed to both unbuffered and buffered MS-222 solutions^16^. Fish even seem to prefer to spend more time in a dark environment than in an illuminated environment containing the anesthetic MS-222, which may indicate that fish would rather undergo discomfort than be exposed to MS-222 (ref. ^17^). Furthermore, the efficacy of MS-222 is variable across animals^13^.

Another euthanasia method that is not universally permitted by law for zebrafish but is often used after special approval by the local authorities is rapid cooling, in which the animals are placed in cold water (2–4 °C). The resulting hypothermic shock is thought to cause death by slowing the rate of cellular activities and neural impulses, finally leading to cardiac arrest^11^. It is mostly considered a humane alternative to an anesthetic overdose, because fish exhibit signs of distress over a much shorter period compared with MS-222-treated animals^16,18^. While the onset of unconsciousness is probably fast^19^, zebrafish larvae survive long periods in cold water^20^. In fact, rapid cooling in larvae younger than 14 dpf requires an exposure of at least 12 h for reliable euthanasia^21^. These results highlight how little is known about the exact timing of death resulting from this method at early stages of development, prolonging the time window in which suffering is possible^22^.

Legislation including the relevant EU directive alternatively permits the application of electricity for euthanasia and refers to this as electrical stunning, even if death, not stunning, is the intended outcome. Electrical stunning protocols have successfully been established for larger fish^23^. However, its suitability has scarcely been investigated for laboratory zebrafish^20^ and electrical stunning has never been successfully established as a humane euthanasia method for zebrafish. Knowledge about reliable parameters including voltage gradient, exposure duration, voltage type, the needed water conductivity and the resulting power density in the fish body are missing^1^. There is also no known adequate and approved equipment available on the market that could be used for this purpose^18^ (see Mocho et al.^20^ for a previous report on electrical stunning^20^). Euthanasia by electrical stunning is most likely to be achieved by asphyxiation of the stunned fish^24^. In small fish larvae with sufficient cutaneous oxygen supply, the mechanism of euthanasia is probably cell death due to unrecoverable damage to the cell membrane integrity or energy depletion of the fish^25^. Larger cells seem particularly susceptible to electric-field-induced membrane damage^26^, which is why the effectiveness of electrical stunning is expected to increase with fish size. Also, different voltage types (such as direct current (DC), pulsed direct current (PDC) or alternating current (AC) and the associated frequencies) are thought to directly influence mortality rate and tissue damage^27–31^. Different aspects of electrical stunning have been investigated for various fish species at different stages of development, but the parameters were often not systematically varied and the effects of electrical stunning on zebrafish behavior and brain activity were not systematically recorded^20,27,32^. To the best of our knowledge, there is not a single study in which potential suffering and stress associated with electrical stunning was quantified in zebrafish, nor in which neural correlates of such experiences were quantified, such as changes to sensory processing induced by the euthanasia method.

Here, we investigated electrical stunning as an alternative humane euthanasia method for zebrafish larvae on both the behavioral and the neurophysiological level using calcium imaging (Fig. 1b,c). We examined and compared different voltage types (DC, PDC and AC) and exposure durations (2 s to 32 s) and their influence on mortality rate and tissue damage. We identified a set of parameters that reliably work at a voltage frequency close to the main frequency of 50 or 60 Hz and for a standard embryo medium conductivity of 670 µS/cm (ref. ^33^), resulting in a 100% mortality rate. Besides behavioral examination we also investigated neuronal activity patterns using calcium imaging, confirming the total loss of coordinated and stimulus-associated neuronal activity and behavior during and after electrical stunning.

## Results

### Mortality rates and behavioral effects of electrical stunning

**Fig. 2:**
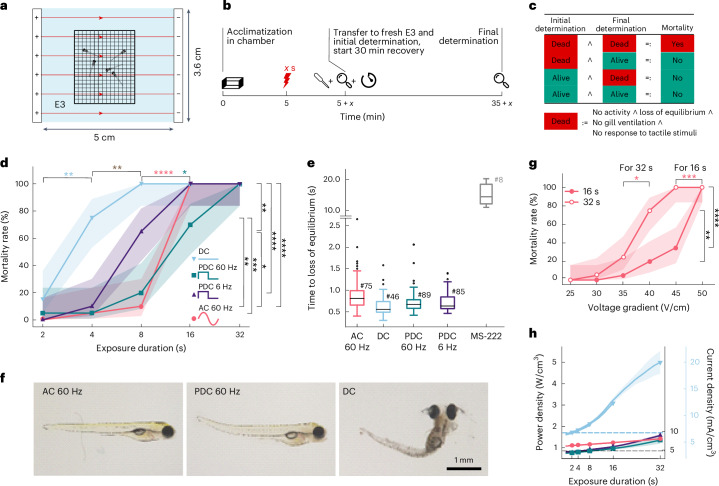
Electric AC fields are well suited for euthanasia of larvae. **a**, A schematic of the experimental setup. **b**, The protocol for assessing mortality. *x* is the exposure duration that was systematically varied over trials. **c**, The logical matrix defining under which condition within the experimental procedure a larva was considered dead. **d**, Mortality rates of different voltage types for different exposure durations at a voltage gradient of 50 V/cm. The asterisks indicate significances by *z*-test for proportions followed by Bonferroni correction between exposure durations for the same voltage type (colored asterisks) and between voltage types for the same exposure duration (black asterisks); *P < 0.05, **P < 0.01, ***P < 0.001, ****P < 0.0001. The envelopes show 95% Wilson confidence intervals. *n* = 400 larvae, *n* = 4 shock exposure trials with 5 larvae each. **e**, The time to loss of equilibrium for different voltage types (animal numbers are provided next to the hash symbols) For comparison, the time to loss of equilibrium for MS-222 (336 mg/l)^22^ is shown in gray. **f**, Morphological effects of electrical stunning at 50 V/cm, 32 s, directly after exposure. **g**, Mortality rates of 16 s and 32 s exposure duration for different voltage gradients at AC voltage. The asterisks indicate significance by *z*-test for proportions followed by Bonferroni correction between voltage gradients and exposure durations; *P < 0.05, **P < 0.01, ***P < 0.001, ****P < 0.0001. The envelopes show 95% Wilson confidence intervals. *n* = 240, *n* = 4 shock exposures with 5 larvae each. **h**, The mean power density and current density of different voltage types for different exposure durations at 50 V/cm. Power and current densities were calculated on the basis of measured currents during the experiment. The dashed lines indicate the expected power density without joule heating (light blue, DC; black, AC and PDC). Light blue *y* axis on the right is for DC and the black *y* axis on the right for AC and PDC experiments. The envelopes show s.d.

To identify the optimal parameter set encompassing voltage gradient, voltage type and exposure duration for electrical stunning, we exposed larvae to DC, AC (60 Hz) or PDC (60 Hz or 6 Hz, duty cycle 50%) electric fields (50 V/cm) in the water bath (Fig. 2a, *n* = 20 larvae per condition; 4 trials with 5 animals per trial). Mortality was assessed as loss of equilibrium, lack of locomotor activity, lack of gill ventilation and lack of responses to tactile stimuli (Fig. 2b,c and Methods). For five different exposure durations (from 32 s to 2 s in logarithmic steps), we conducted four independent trials involving five larvae each. Different mortality rates occurred at different exposure times depending on the type of voltage used. At 32 s of exposure, all voltage types resulted in 100% mortality. For 16 s of exposure, only 60 Hz PDC was not able to reach a 100% mortality rate. At 8 s of exposure, significant divergence in mortality rates among voltage types emerged for the first time (*z*-test for proportions, *P* < 0.01), with rates dropping below 20% for all types at 2 s (Fig. 2d). Behavioral loss of equilibrium was immediate across all voltage types, occurring within 900 ms (AC, 0.89 s ± 0.38 s; DC, 0.64 s ± 0.25 s; PDC 60 Hz, 0.73 s ± 0.28 s; PDC 6 Hz, 0.72 s ± 0.22 s (average ± s.d.)), suggesting an instant onset of loss of sensation (that is, anesthesia) (Fig. 2e)^34^.

Depending on the voltage type used, several morphological changes, including body curvature, opaque brain tissue and sometimes fractures, could be observed for longer exposure durations (32 s and 16 s) for all voltage types. Morphological abnormalities were recorded directly, 15 and 30 min after exposure. These included visible destruction of tissue, skin and eyes as well as severe deformation of the body axis (lordosis, kyphosis and scoliosis) for DC and 6 Hz PDC and only up to medium-grade body axis deformation for AC and 60 Hz PDC (Fig. 2f and Supplementary Fig. 1). Many AC-treated larvae showed no morphological phenotype directly after stunning and only developed a phenotype over the course of 30 min. None of the larvae exhibiting abnormalities survived.

Because AC voltage and exposure durations of 16 and 32 s achieved 100% mortality with a fast onset and only minimal morphological abnormalities, we next refined these parameters with regard to the voltage gradient. We tested five different voltage gradients (from 45 V/cm to 20 V/cm) for 16 s and 32 s exposure duration. Already for 45 V/cm, mortality rates differed significantly, and only the 32 s trial still reached 100% mortality rate (*z*-test for proportions, *P* < 0.0001) (Fig. 2g). We therefore concluded that AC (60 Hz), 50 V/cm and 32 s is a reliable and effective parameter set to achieve 100% mortality rate, and it was used for all subsequent calcium imaging experiments to assess the effects of electrical stunning on the neurophysiological level. To extend our observation period and confirm that no larvae recover, we conducted another, independent experiment using our optimal parameter set (AC, 60 Hz, 50 V/cm, 32 s) and we included one additional observation time point in the morning of the day following the electrical stunning. Again, all larvae were dead after the 30 min recovery period. None of the larvae came back to life overnight and none of them showed a heartbeat the next morning (*n* = 20 larvae, 4 trials with 5 fish each).

### Electrolysis and joule heating

During experiments using the exposure chamber, electrolysis could be observed as small bubbles formed at the electrode surfaces. Because the pH of the E3 medium remained stable at 7 after electrical stunning, this was not investigated further. However, electrolysis effects should be explored more comprehensively for a final implementation of electrical stunning.

Furthermore, an increase in temperature and, thus, in electrical current was observed during exposure to AC, DC and PDC for all exposure durations. Temperature increases ranged from 1.26 ± 0.33 °C for PDC (60 Hz) and 2 s exposure duration to 24.25 ± 0.22 °C (average ± s.d.) for DC and 32 s exposure duration (Supplementary Fig. 2). The temperature rise was 13.3 ± 0.53 °C for AC (60 Hz), 50 V/cm, 32 s, with 36.1 °C being the maximum temperature reached. The observed change in temperature is due to joule heating, where an electric current heats a medium. In turn, the conductivity changes, which again influences the current magnitude. These effects lead to a rise in power density in the exposure chamber, which in principle could have influenced the mortality rates (Fig. 2h). No rise in temperature was observed during calcium imaging experiments (see below), where a single larva was agarose-embedded between two small electrodes. This is most likely due to the substantially smaller electrode surfaces and the proportionally larger E3 volume in the Petri dish, which serves as a heat sink. To rule out that the temperature rise and increased power density affect mortality rates for AC at 50 V/cm and 32 s exposure duration, and to ensure comparability between the mortality rate experiments using the exposure chamber and the calcium imaging experiments not using it, a transfer experiment was carried out where single agarose-embedded larvae were exposed to an AC field with 50 V/cm for 32 s using the small electrodes (Methods). After exposure, larvae were freed from agarose and mortality was determined using the same behavioral criteria as used for free-swimming experiments. No difference in mortality rate was observed (*n* = 6 larvae with 100% mortality rate). Thus, even in the absence of a rise in power density associated with the exposure chamber, 100% mortality can be achieved with AC at 50 V/cm and a 32 s exposure duration.

### Swim bursts during electrical stunning

**Fig. 3:**
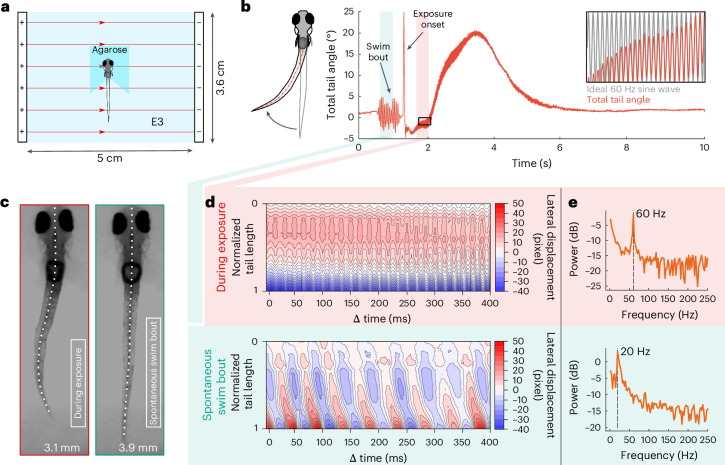
The AC field drives direct muscle contractions. **a**, A schematic of the experimental setup. Agarose surrounding the larva’s tail has been removed. **b**, Left, a schematic of larva illustrating tail displacement during electrical stunning. The orange arrows indicate the skeleton used for computing the total tail angle. Right, the total tail angle during exposure. The arrows indicate a spontaneous swim bout and the exposure onset. The inset shows the tail angle during exposure, which perfectly matched a 60 Hz sinusoidal wave. **c**, The length of the larva during (left) and before (right) electrical stunning. **d**, Top, the lateral tail displacement profile during electrical stunning. Bottom: the lateral tail displacement profile during the spontaneous swim bout. **e**, Top, the power spectrum during electrical stunning. Bottom, the power spectrum during the spontaneous swim bout. *n* = 2.

Within the first seconds of exposure to any tested electrical field, we observed short bursts of tail movements, either during or after loss of equilibrium (Supplementary Video 1). If this corresponded to a planned locomotion behavior, this might have implications regarding the state of the animal and associated potential of suffering. To rule out any such kind of active locomotion, we recorded a high-speed video of a single embedded larva with its tail cut free and exposed to an AC field at 50 V/cm for 32 s (Fig. 3a). A kymograph of the total angle of the tail revealed a strong initial tail deflection at exposure onset followed by a tail beat frequency perfectly matching the 60 Hz sinusoidal frequency of the electric field applied, strongly suggesting that the observed muscle movements were passively driven by the electric field (Fig. 3b,e, top and Supplementary Video 2). Furthermore, both tail beat amplitude and standard length^35^ decreased during exposure (preexposure 3.9 mm, intraexposure 3.1 mm; Fig. 3c), presumably as a result of bilateral tail muscle contraction driven by the electric field. Also, the spatiotemporal curvature pattern of the tail differed drastically for tail undulations during exposure as compared with a spontaneous swim bout (Fig. 3d). During the spontaneous swim bout, the point of maximal curvature traveled along the tail in rostrocaudal direction for each tail undulation at a frequency of 20 Hz (Fig. 3e, bottom), which is conducive to forward swimming^36^. However, during electric field exposure, the stereotypic spatiotemporal pattern could not be observed, that is, the complete tail contracted at once in an abnormal, bilaterally synchronized fashion.

The observed phenomenon might be related to the pseudo-forced swimming described previously^37^, although the voltage type used in that study was DC.

### Loss of visually driven neural activity during electrical stunning

**Fig. 4:**
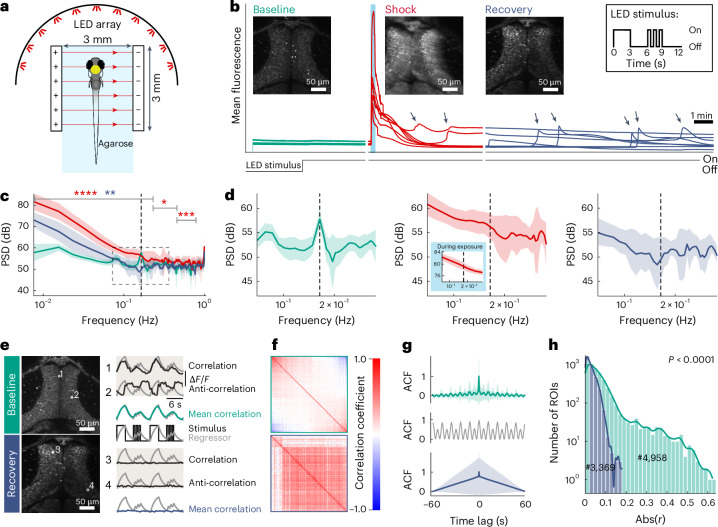
Absence of sensory-evoked neural activity during and after electrical stunning. **a**, A schematic of the experimental setup positioned under the two-photon microscope. The yellow dot indicates the imaged brain area (optic tectum). **b**, Mean calcium signals for baseline, shock and recovery recording for all fish (one trace per fish). The light-blue bar indicates exposure duration. The LED stimulus trace indicates when a visual stimulus was used or not. The arrows highlight the occurrence of slowly propagating calcium waves occurring after electrical stunning. **c**, The mean PSD for baseline, shock and recovery recording (green, red and dark blue as used in **b**). The envelopes show s.d. The dashed line indicates the visual stimulus main frequency of 0.1667 Hz. The asterisks indicate periods with significant differences to baseline recording (two-way repeated-measures ANOVA followed by Tukey’s HSD test; *P < 0.05, **P < 0.01, ***P < 0.001, ****P < 0.0001). **d**, The inset of **c** for baseline, shock and recovery recordings. **e**, Example calcium traces of neurons (black) (anti-)correlated with the stimulus (gray), and mean correlation traces (green and dark blue) of 20% best correlated ROIs for baseline and recovery recording. **f**, ROI cross-correlation matrices for baseline and recovery recording (top and bottom). **g**, The mean ACF for baseline and recovery recording (top and bottom). The envelopes show s.d. Gray shows the ACF for the stimulus (regressor) itself. **h**, A histogram of absolute ROI cross-correlation coefficients (Abs(*r*)) for baseline and recovery recordings. Hash symbols indicate the overall number of neurons (ROIs) used. *P* value derived from Levene test. *n* = 7.

To quantify and compare neuronal activity before, during and after electrical stunning, two-photon calcium imaging was performed while applying a simple and strong visual on–off stimulus (full-field flash) and simultaneously exposing larvae to an electric field (AC, SIN, 60 Hz, 50 V/cm, 32 s; Fig. 4a). Recordings were done in the optic tectum, which is known to be a hub in visual processing and contains different populations of neurons well responding to on and off flashes^38,39^. Because the visual system of zebrafish larvae develops rapidly, strong optokinetic responses and associated sensory-driven neural activity are already established by 4 dpf (refs. ^40,41^). The absence of such sensory-driven activity can thus serve as a reliable indicator of defective neural processing.

Mean fluorescence time courses across all pixels obtained from the calcium recordings before (baseline), during (shock) and after (recovery) electrical stunning revealed drastic changes in activity patterns and overall intensities (Fig. 4b). Exposure to the electrical field also gave rise to a fast-propagating calcium wave (<2 s) with high mean fluorescence intensities along with strong spatial drifts (>20 μm), resulting in permanent morphological changes that impeded consistent segmentation of individual neurons across recording time (Supplementary Video 3). After exposure, mean fluorescence intensity across each image frame decreased until the occurrence of further, slowly (>2 s) propagating calcium waves, with different characteristics than the initial wave (Fig. 4b). Due to these strong changes in fluorescence intensity, adjustment of laser power and photomultiplier tube (PMT) gain between individual recordings was necessary to avoid overexposure and damage to the PMTs. Thus, absolute fluorescence intensities cannot and should not be compared across recordings.

To investigate whether visual processing still occurred during and after electrical stunning, the mean power spectral density (PSD) of all pixels within a recording was analyzed across all fish, where for intact vision-related processing the stimulus main frequency (0.1667 Hz) should not be affected much by spatial drifts of the larva in the recording window (Supplementary Fig. 3). As expected, PSD analysis revealed a total loss of the visual stimulus main frequency (0.1667 Hz) during shock and recovery (*P* < 0.01 between 0 Hz and 0.22 Hz for shock and recovery recording, two-way repeated-measures analysis of variance (ANOVA) followed by Tukey’s honest significant difference (HSD) test; Fig. 4c,d). A further PSD analysis covering only the period during exposure to the electric field (32 s) confirmed this observation (Fig. 4d, middle, inset). Furthermore, the mean power density strongly increased toward lower frequencies during shock and recovery recordings, representing the observation of brain-wide, slowly changing calcium intensity.

Next, we segmented neurons in each the baseline and recovery recording to ask how the population tuning to the visual stimuli changed. The optic tectum contains diverse sets of neurons, some correlated and others anti-correlated or not correlated with our luminance stimulus. Also, on average, fluorescence time courses of neurons should inherit some form of periodicity as the stimulus itself consisted of various periodic parts. By contrast, during and after exposure, all these characteristics should have vanished to confirm loss of intact neural processing and the effectiveness of electrical stunning as a euthanasia method. In total, 4,958 segmented regions of interest (ROIs) were analyzed during baseline recording and 3,369 ROIs during recovery (*n* = 7 fish). The difference in number of detected ROIs is due to the technical difficulty of detecting ROIs during recovery. Neurons in the optic tectum showed diverse activity patterns during baseline recording with regard to visual stimulation, where 3.5% of the overall number of neurons were correlated or anti-correlated to the visual regressor with a correlation coefficient above 0.3 (Fig. 4e,h). During recovery recordings, not a single neuron had a correlation coefficient higher than 0.3. Also, a Levene test revealed a significant difference in the variance of correlation with the visual regressor between both recordings (*P* < 0.0001). To characterize the stimulus-related activity, we quantified the pairwise cross-correlation between the 20% best correlated neurons within each fish for both baseline and recovery recording (Fig. 4f). Neurons within each matrix were sorted by their correlation coefficient from positive to negative, resulting in a structure with two visible anti-correlated populations (red and blue) during baseline recording. These populations correspond to neurons responding to either the on or off phase of the visual stimulus. No similar structure could be found during the recovery recording, where almost all neurons correlated with each other, which could be explained by an overall synchronous decrease in fluorescence due to the fading out of the initial calcium wave. Finally, an autocorrelation function (ACF) of both recordings yielded a perfect periodic pattern for the baseline recording representing the periodic stimulus-related activity pattern, and nothing but linear decrease during the recovery recording (Fig. 4g).

All these results strongly indicate that, during electrical stunning and afterward, no neural processing with respect to visual perception was possible, as the dominance of monotonous calcium signals indicates nonintact brain activity and presumable loss of sensation.

## Discussion

In this study, we investigated electrical stunning as an alternative euthanasia method for zebrafish larvae. We systematically evaluated different voltage types, voltage gradients and exposure durations, resulting in a robust parameter set that can be implemented under real-world conditions (32 s, 50 V/cm, AC, 100% mortality). We further demonstrated that observed tail movements during exposure are unrelated to any kind of voluntary locomotion and that electrical stunning provokes an immediate loss of coordinated neuronal activity—and, therefore, probably suppresses any sensation that could arouse suffering. Importantly, larvae never recovered from this terminal state, and their rapid loss of equilibrium confirms that electrical stunning minimizes the remaining time window before complete incapacitation, especially compared with previously established euthanasia methods.

### Reliable parameters are straightforward to implement

One of our major aims was not only to comprehensively examine the effects of electrical stunning on behavioral and neurophysiological levels but also to devise a reliable parameter set that can be implemented in practice across international and disciplinary boundaries. Therefore, an important consideration was to also examine a sinusoidal AC voltage with a frequency near 50 or 60 Hz, as is common in many countries. While both 32 s and 16 s exposures lead to 100% mortality at 50 V/cm (Fig. 2d), we recommend 32 s to account for possible variations in specific future implementations. We confirmed the loss of stimulus-associated neural activity only for a 32 s exposure and not for a 16 s exposure, posing a limitation to the 16 s. Despite joule heating raising the resulting power density to 1.4 W/cm^3^ after 32 s during behavioral assays (Fig. 2h), the calcium imaging experiments showed that 0.85 W/cm^3^ is in fact sufficient to reliably euthanize zebrafish larvae. This is in accordance with published findings that a calculated power density of 0.7 W/cm^3^ with PDC at 50 Hz, 25 V/cm and 1120-1130 µS/cm (ref. ^20^) is sufficient to euthanize zebrafish larvae, whereas a power density of 0.00086 W/cm^3^ using AC at 12.5 V/cm and 2000 µS/cm (ref. ^42^) has no influence on the survival rate. Although the conductivity of small fish species is thought to be less than 30 µS/cm (ref. ^43^) and, therefore, the conductivity of a zebrafish larva is most likely even smaller, our medium for electrical stunning with a conductivity of 680 µS/cm was chosen specifically to be consistent with a standard E3 embryo medium (5 mM NaCl, 0.17 mM KCl, 0.33 mM CaCl_2_, 0.33 mM MgSO_4_ and 10^−5^% methylene blue) as it is commonly used for zebrafish larvae^33^.

The suggested parameters should be easy to implement in an electrical stunning setup owing to their characteristics, and the principle layout of the exposure chamber introduced in our experimental setup (Fig. 1b and Supplementary Fig. 4) might provide a possibility to build and establish safe and adequate devices as required by the EU directive 2010/63/EU^18^.

### Electrical stunning reduces time window to onset of euthanasia

We have demonstrated that loss of equilibrium can be reliably induced within a single second using electrical stunning, which is an important aspect when evaluating an euthanasia method^44^. On a behavioral level, an animal is likely to be unconscious from the time point on its losing its righting reflex (which in our study is equivalent to the loss of equilibrium)^34^. Tricaine (MS-222) is known to work relatively slowly in this respect, as it takes between 25 s and 34 s for adult zebrafish to lose equilibrium at a concentration of 250 mg/l (refs. ^15,16,45^). Even at 336 mg/l it takes about 15 s for larvae until the loss of the righting reflex^22^. Although for rapid cooling the time window to onset decreases to less than 15 s for larval zebrafish, a constant exposure to cold is still required for 12 h to ensure reliable euthanasia^19,21^. The much faster onset time (0.89 s ± 0.38 s, average ± s.d. for AC, 50 V/cm) for electrical stunning therefore has an immediate effect on the reduction of any possible stress and suffering. Aversive reactions shown by larvae as reported for tricaine use^20^ were not observed.

### AC is the best-suited voltage type for electrical stunning

Depending on voltage type and voltage gradient, different injuries such as tissue disruption, vertebral dislocations and even fractures are known to occur in fish. These injuries most likely arise from strong muscle contractions caused by direct electrical stimulation of either muscles or the neuromuscular pathway and are influenced by the voltage type used as well as the voltage gradient and frequency^23,46,47^. There are different findings regarding how different types of voltage affect mortality and injuries in fish. According to ref. ^31^, using DC or reducing the frequency at PDC results in less injury to fish whereas AC is most likely to cause tetany, potentially resulting in injury and mortality. Also, ref. ^48^ reported that spinal injuries have mostly been associated with AC, and there are numerous reports about non-DC waveform that together with different frequencies negatively affected injury and survival rates^23,47–50^. However, this does not seem to be the case for embryos and zebrafish larvae, which are found to be more vulnerable to DC^27,28,42,51^. This concurs with our findings, where DC (50 V/cm, 32 s) led to severe tissue destruction and ruptures (Fig. 2f). DC treatment was also associated with the highest reached temperatures due to joule heating (up to 46.9 ± 0.4 °C), which probably contributed to the morphological defects of larvae via thermal denaturation of proteins. During treatments with AC, however, the temperature increased only mildly and stayed below 38 °C (50 V/cm, 32 s, AC 35.03 ± 0.77 °C), a temperature that is frequently used for live zebrafish in genetic experiments^52–55^, while only up to medium body axis deformations could be observed. However, further studies are needed to ascertain whether cells and tissue of AC-euthanized larvae remain intact and useable for any postmortem analyses. Although the DC-associated injuries and body deformations are unlikely to impact larval welfare relative to AC, given DC’s loss of equilibrium also occurring in within 1 s (0.64 s ± 0.25 s), the observable injuries might misconstrue electrical stunning as an unsuitable euthanasia method. To avoid this issue and taking into account that a good euthanasia method should also consider the emotional effect on observers and operators^44^, we endorse AC as the most compelling voltage type for electrical stunning applications on zebrafish larvae.

### Electrical stunning with AC is followed by widespread propagating calcium waves

Over the course of investigating neuronal activity during and after electrical stunning, we observed a strong synchronous rise of intracellular calcium (also referred to as a fast-propagating calcium wave) alongside the onset of the electrical field as well as concomitant morphological changes. Most likely, this first wave was caused by the influx of calcium from extracellular space driven by the electric field^56^. This influx might in turn have led to ATP depletion and mitochondrial failure due to the loss of membrane potential, further causing imbalance of calcium and potassium and, finally, cell death^57–60^. The exact mechanism underlying cell and organismal death in zebrafish larvae via AC-based electrical stunning remains unclear. Several (interacting) cell death pathways have been discussed in a recent review on the effects of electroporation^25^. Although we did not characterize the differences between the initial fast-propagating and following slow-propagating calcium waves in detail, they seemed to differ in propagating speed, maximum calcium intensity and place of origin. The slow-propagating calcium waves (as seen by eye) might be similar to earlier observations in zebrafish larvae, where a slow wave occurred after cardiac arrest, traveling in a caudal–rostral direction and thought to be associated with neuronal death and brain damage^61^. Other brain-wide calcium waves in zebrafish larvae, akin to what is called a spreading depolarization^62,63^, could be observed in the context of heat-induced neural dysfunctions^64^. In rats, the presence of a large-amplitude electroencephalography wave after decapitation has been described. It was assumed that the wave reflects a massive opening of ion channels (depolarizing wave) that occurred owing to neurons lacking energy, which might have led to a breakdown of transmembrane potential, leading to oscillations and the observed wave^65,66^. Although a comprehensive elucidation of the mechanism and relevance of these slow-propagating calcium waves necessitates further investigation, our findings, alongside perception of visual stimuli and behavior of larvae during and after electrical stunning, argue against their association with suffering or stress.

### Electrical stunning is an effective alternative euthanasia method

In summary, our results suggest that electrical stunning, when using the proposed parameters, is an effective euthanasia method for zebrafish larvae. Although only examined for 4 dpf in this study, the suggested parameter set is most likely to also reliably work for older zebrafish larvae, as vulnerability of fish to electrical current is thought to increase with body size^26^. Besides immediate loss of equilibrium, the irrevocable loss of coordinated and sensory-driven neuronal activity strongly indicate rapid onset of unconsciousness during treatment^34^. As far as we know, the only other studies examining neuronal activity alongside exposure of electrical fields to fish were performed applying electroencephalography on African catfish and cichlids. In these studies, a general epileptiform insult could be observed, which was followed by fibrillation after electrical stunning^67,68^. The slowly propagating calcium waves after electrical stunning observed in this study encourage further investigation, but they most likely do not influence the efficacy of the euthanasia method itself or the animal’s welfare. Given the fact that electrical stunning is already a permitted euthanasia method for fish in the EU, its implementation in laboratory routines could plausibly contribute to a refinement in the sense of 3R (replacement, reduction and refinement)^4,69^ across many disparate research labs.

## Methods

### Animals

Animal experiments were permitted by the local government authorities (Regierungspraesidium Tuebingen) and conducted in accordance with German federal law and Baden-Württemberg state law. Zebrafish larvae (*Danio rerio*) were reared on a 14/10 h light/dark cycle at 28 °C in E3 medium (5 mM NaCl, 0.17 mM KCl, 0.33 CaCl and 0.33 MgSO_4_) with 0.01% methylene blue. Wild-type (nacre +/−) larvae were used for the mortality rate and behavioral experiments. Transgenic zebrafish expressing GCaMP6f [*Tg(HuC:H2B-GCaMP6f)jf7Tg*] were used for the two-photon calcium imaging experiments. The top 10–20% of larval zebrafish with high calcium indicator expression levels were selected from all fish at 3 dpf for in vivo calcium imaging experiments using an epifluorescence stereoscope. All behavioral and calcium imaging experiments were performed on 4 dpf zebrafish larvae at room temperature. Supplementary Tables 1–3 contain lists of experimental groups and animals used per group.

### Exposure chamber for mortality rate experiments

To determine mortality rates of multiple free-swimming larvae, an exposure chamber covering a volume of 50 × 36 × 15 mm was designed and built out of polystyrene. The internal dimensions of the exposure chamber represent a trade-off between the space needed to safely expose multiple larvae to an electric field and the required voltage gradients. To protect against leakage currents, the programmable power source GW Instek ASR2050R (Good Will Instruments) was connected to the exposure chamber by a residual-current circuit breaker (F200, B Type, ABB Ltd (Asea Brown Boveri)). This guaranteed safe operation for both AC and DC voltages. Two stainless-steel electrodes (36 × 15 mm) with a thickness of 1 mm were placed inside the chamber at a distance of 50 mm. A custom-made 3D-printed electrode holder was used to hold the electrodes in place and to ensure the exact distance of 50 mm. This electrode holder also included a rigid net with an aperture of no more than 405 µm (PA-405/47, Eckert), in which larvae could be placed during exposure. This ensured that larvae were always exposed to the center of the electric field (Supplementary Figs. 4 and 5a). Electrodes were constructed by the precision mechanics workshop of Eberhard Karls University, Tuebingen, Germany. All polymer components were constructed and 3D-printed in the lab (Ultimaker3, Ultimaker B.V., ink: Ultimaker Material PLA Black, diameter 2.85 mm). For safety reasons, operation of the exposure chamber depends on a snap-action switch that requires a closed lid. The lid and bottom of the chamber are transparent to enable diffuse back illumination at 920 nm from below and behavioral recording from above. A light-emitting diode (LED) array containing 48 LEDs with a 50° emission angle and a diffuser (frosted glass) was used for illumination. Recording was done at up to 30 Hz using a camera (DMK23UV024, The Imaging Source Europe GmbH) with a 6 mm objective and an infrared (IR) longpass filter (SCHOTT RG780, Edmund Optics). Both the power source and the camera recording were controlled by the custom-written Python software ‘synchASR2000-control’^70^. The software allowed us to individually set voltage type (DC, PDC and AC), overall voltage, maximum current, frequency and exposure duration.

### Determining mortality rates

For each experiment, the exposure chamber was filled with 24 mL of room-temperature E3 medium (680 µS/cm conductivity). Five larvae were transferred into the chamber, followed by a 5 min acclimatization period. After that period, the temperature of the E3 medium inside the chamber was measured once using a digital thermometer (GMH 3200 Series, Greisinger) and the lid was closed. An electric field was then applied for a specific exposure duration. In the case of controls, no electric field was applied. During the exposure, larvae were video recorded. The recording was synchronized with the onset of the electric field via the Python software using a timestamp. Immediately after the exposure, the lid was opened and the temperature was measured again. Larvae were then moved into a dish of fresh E3 (room temperature). To determine mortality rates, all larvae were visually examined via a stereo microscope directly after exposure to an electrical field and a second time after a 30 min recovery period in fresh E3 (Fig. 2b). Mortality was defined by the following criteria: no activity of any kind, loss of equilibrium, no operculum movement and no startle response (tested by a strong tactile stimulus in the form of poking with a blunt metal needle). Together with muscle tone and cardiac failure (which were not assessed here), these criteria correspond to the fourth stage of anesthesia in fish, describing overdose^71^. Muscle tone observation was excluded owing to the ability of electrical fields to cause muscle tension. Meanwhile, cardiac arrest is not a suitable criterion for death in larval zebrafish, because their small size ensures that oxygen diffusion into vital tissue is sufficient for survival even after the heart stops beating^11^. Heartbeat may persist for up to 10 min after euthanasia with MS-222 and 40 min after hypothermic shock^11^. Some studies even report that MS-222-treated larvae were able to recover their heartbeat when subsequently transferred to fresh water^11,13^. Also, in our study, cardiac arrest rates (as assessed via visual inspection using a stereoscope) differed from mortality rates for exposure durations between 2 s and 32 s (Supplementary Fig. 6). Larvae were considered dead only if all tested criteria were met at both two observation points (directly after exposure and after the 30 min recovery period) (Fig. 2c). After the second observation point, all larvae were put into ice water for 20 min and were then transferred into a freezer where they were kept for at least 48 h to ensure proper euthanasia for potentially survived fish. In total, 600 fish were used in 30 different groups, with each group containing 4 independent trials with 5 fish each (Supplementary Table 1). Significant differences between different voltage types and between exposure durations within each voltage type were determined by *z*-test for proportions followed by Bonferroni correction. To further minimize potential stress and suffering and to ensure animal welfare, a pilot trial with 20 larvae anesthetized with MS-222 (168 mg/l) and 20 nonanesthetized larvae exposed to 50 V/cm for 32 s (DC) was conducted before all experiments. Because results revealed no increased mortality rates for MS-222 and no visible difference in animal behavior during exposure, all subsequent experiments were carried out without anesthetic (*z*-test for proportions, *P* > 0.05; Supplementary Fig. 7).

### Determining loss of equilibrium

Loss of equilibrium was determined by hand using the video recordings from the mortality rate experiments and the image processing package Fiji^72^. The time difference between the exposure onset of electrical stunning and the first frame where larvae departed from the upright position was measured. Not all recorded larvae from the mortality experiments could be analyzed, because they sometimes swam into the shadow cast by the electrode frame, which made it impossible to observe the time point of loss of equilibrium. Therefore, the number of larvae analyzed in this regard differed from the total number of larvae recorded. In total, 295 fish were analyzed.

### Setup for simultaneous calcium imaging and electrical stunning

For calcium imaging experiments, a two-photon IR laser (Coherent Chameleon Vision S, Coherent) at a wavelength of 920 nm was used and calcium signals were recorded using a movable-objective microscope (MOM; Sutter Instruments) with GaAs PMTs, C7319 Hamamatsu preamplifiers and the MScan software (2016 version) by Sutter. All recordings were conducted in the optic tectum, scanning a single plane per fish approximately 40–70 µm deep with a 20×/1.0 objective (Zeiss W Plan-Apochromat 421452-9800) at a frequency of 2 Hz and a magnification of 1.8×. To conduct calcium imaging alongside simultaneous visual stimulation and exposure to an electric field, a setup containing a LED ring with 18 × 650 nm LEDs (EVERLIGHT Electronic, EVL 383-2SDRD/S5, 125 mcd) and a centered 50 mm Petri dish with two small stainless-steel electrodes was developed. The electrodes (2 × 3 mm, 0.5 mm thick) were placed at a distance of 3 mm in the Petri dish using a custom-made 3D-printed frame. The frame enclosed the electrodes from three sides to avoid any unwanted possible contact with the microscope objective (Supplementary Figs. 5c and 8). To connect the electrodes to the power source, a 200-µm-thin copper plate was welded to their back sides to allow soldering of cables. The power source was controlled by the custom-written Python software ‘synchASR2000-control’. To present a strong visual on/off stimulus, the LED ring was connected to an Arduino NANO, controlling the power supply of the LEDs. To gate the LEDs during laser scanning, the Arduino NANO considered the fly-back signal provided by the MOM. On/off signals from the LEDs, fly-back signal from the MOM and the onset signal of the electrical field were recorded at 100 Hz via an Arduino UNO and another custom-written Python software called ‘recSignals-control’. The whole setup was electrically separated from the microscope objective by a modified SM1L05 lens tube containing an insulating plastic spacer.

### Calcium imaging alongside electrical stunning and visual stimulation

For all calcium imaging experiments, the following shock parameters were used: 32 s, 50 V/cm, AC, SIN. For each experiment, a single zebrafish larva was embedded in 1.6% agarose gel (Biozym Sieve GeneticPure Agarose 850080 mixed with E3 medium) between the electrodes (Fig. 4a). The embedding was performed in the absence of any anesthetic drugs. Agarose is electrically neutral, so the electrical conductivity of the gel is determined by the liquid added^73^. Therefore, it can be assumed that the conductivities of E3 medium and agarose gel were approximately the same and it was unlikely to affect the homogeneity of the electric field. Each experiment consisted of three subsequent recordings: baseline recording (6 min, 3 min of which with visual stimulation), shock recording (6 min with visual stimulation) and recovery recording (12 min with visual stimulation) (Fig. 4b). Recordings were done in the optic tectum, which is known to be a hub in visual processing and contains different populations of neurons responding well to on and off flashes^38,39^. In total, seven fish were imaged.

### Swimming burst analysis

A high-speed recording (IDT iN8-S1 camera) with a sampling rate of 500 Hz was performed on a single agarose-embedded 4 dpf larva (nacre +/−) using the exposure chamber without the rigid net (Fig. 3a). Agarose was removed around the tail to observe exposure-related movements, as described in Arrenberg^74^. Recording was performed for 10 s (exposure onset was approximately 1 s after the start of recording). Electrical stunning was performed with a sinusoidal AC field, 60 Hz, 50 V/cm, for 32 s.

To analyze the recordings, images were first smoothed using a median filter (filter size 20 × 20 pixels) to remove noise. Afterward, the gray values of all pixels were first inverted and then binarized. Areas smaller than 600 pixels in size were removed to clear the image, leaving only the silhouette of the fish. A skeleton of the silhouette representing the spine of the fish was computed to track tail deflections. Lateral displacement of the tail was defined as the distance (in pixels) of each skeleton pixel to the fish’s original rostrocaudal axis, normalized to the mean displacement during the spontaneous swim bout and field exposure. The total tail angle (in degrees) was calculated for each frame by computing the angles between all neighboring pixels on the skeleton and then taking the sum along the entire tail. Tail length was normalized to the shortest skeleton computed using 1D interpolation. In total, two fish were recorded and analyzed.

### Transfer experiment

To compare results across experiments on free-swimming and embedded larvae, a transfer experiment was conducted. A single larva was embedded in 1.6% agarose between the small electrodes in the setup for simultaneous calcium imaging and electrical stunning, and a shock (32 s, 50 V/cm, AC) was applied. Directly after shock application, the larva was carefully freed, and mortality was assessed using the behavioral criteria described above. In total, six fish were examined.

### Analysis of ROIs and cross-correlation matrices

All data analysis was done in Python. Baseline and recovery recordings were registered to s.d. *z* projection, and ROIs were segmented using suite2p^75^. The calcium signal (Δ*F*/*F*_b_, that is, fluorescence changes relative to baseline) at time point *t* was calculated as in ref. ^22^. The calcium signal of each ROI was calculated for the second half of the baseline recording (visual stimulus present) and during the period preceding onset of a slow-propagating wave for the recovery recording, which differed across trials. The overall number of ROIs for baseline and recovery recording differed, which could be explained a combination of morphological changes during electrical stunning, the overall decrease in calcium activity and the increase of synchronized activity among ROIs that led to poor segmentation results in the recovery recordings.

Regression-based identification of stimulus-encoding neurons was used in this study to quantify alterations of vision-related activity during and after electrical stunning^76^. For this purpose, an ‘on’ regressor was computed by first resampling the recorded on/off signals of the visual stimulus to 2 Hz and then convolving them with a calcium impulse response function using an exponential decay time constant of 1.61 s for GCaMP6f. ROIs were then correlated with the on regressor to test how well their activities matched the expected calcium signal of a neuron responding to light onsets. Distributions of correlation coefficients for baseline and recovery recording were analyzed via a Levene test.

To generate cross-correlation matrices, ROIs were sorted by their correlation coefficient (from positive to negative correlation with the stimulus) and a cross-correlation matrix of the 20% best correlated ROIs was computed. Matrices of all fish were brought to the same size (mean size of all matrices) using linear interpolation. A weighted average matrix was then computed, based on the total number of ROIs per fish.

### PSD analysis

Welch’s PSD method was used for estimating the power density of fluorescence at different frequencies for all recordings (baseline, shock and recovery). Because recordings during exposure to an electrical field were affected by strong spatial drifts of the preparation and image registration and across-frames segmentation of ROIs were therefore not possible, the PSD for each pixel in each recording for all fish was calculated, using the entire recording length of each raw image time series without any preprocessing. Calcium traces for all pixels were first detrended by subtracting the mean, then PSDs were calculated. Finally, the mean PSD spectrum of all pixels was calculated for all recordings. To prove that a large drift in the recording will not affect the mean PSD spectrum in a major way, and that the most prominent frequencies will still be visible, a 100-pixel drift over 720 frames (6 min) was simulated for a stable baseline recording and PSD was calculated subsequently (Supplementary Fig. 3). PSD results were analyzed via two-way repeated-measures ANOVA with Tukey’s HSD test.

### Reporting summary

Further information on research design is available in the Nature Portfolio Reporting Summary linked to this article.

## Online content

Any methods, additional references, Nature Portfolio reporting summaries, source data, extended data, supplementary information, acknowledgements, peer review information; details of author contributions and competing interests; and statements of data and code availability are available at 10.1038/s41684-024-01505-0.

## Supplementary information

Supplementary Information Supplementary figures, animal numbers and illustrations of experimental setups

Reporting Summary

Supplementary Video 1 Video of freely swimming larvae during exposure to electrical field. Animals lost equilibrium directly after the electrical field had been applied. Two animals also showed short bursts of tail movements, which were passively driven by the electric field.

Supplementary Video 2 Before the electric field exposure, this animal performed a spontaneous swim bout. During electric field exposure, the tail was passively driven by the electric field for a few seconds. Note the differences in tail beat frequency, curvature pattern of the tail and tail length.

Supplementary Video 3 Calcium image time series of a single optical slice in the optic tectum. Upon electric field application, a fast-propagating calcium wave occurs and morphological changes lead to image drift.

Supplementary Data 1 Surface geometry stereolithography stl files for 3D-printing electrode frame and LED frame for the 2p setup.

Supplementary Data 2 Surface geometry stereolithography stl files for 3D-printing electrode frame and exposure chamber for the free-swimming experiments.

## Data Availability

All data from this study are available from the corresponding author upon request.
