# Supplementary material for: Behavioral and neurophysiological effects of electrical stunning on zebrafish larvae
Source: Lab Anim (NY). 2025 Jan 27;54(2):50–8. doi: 10.1038/s41684-024-01505-0 (PMC11790490; doi:10.1038/s41684-024-01505-0)
Supplement: Supplementary file 1 — Supplementary figures, animal numbers and illustrations of experimental setups [file 41684_2024_1505_MOESM1_ESM.pdf]

https://doi.org/10.1038/s41684-024-01505-0

Behavioral and neurophysiological effects of electrical stunning on zebrafish larvae

In the format provided by the authors and unedited

Supplementary Figures

Supplementary Figure 1:

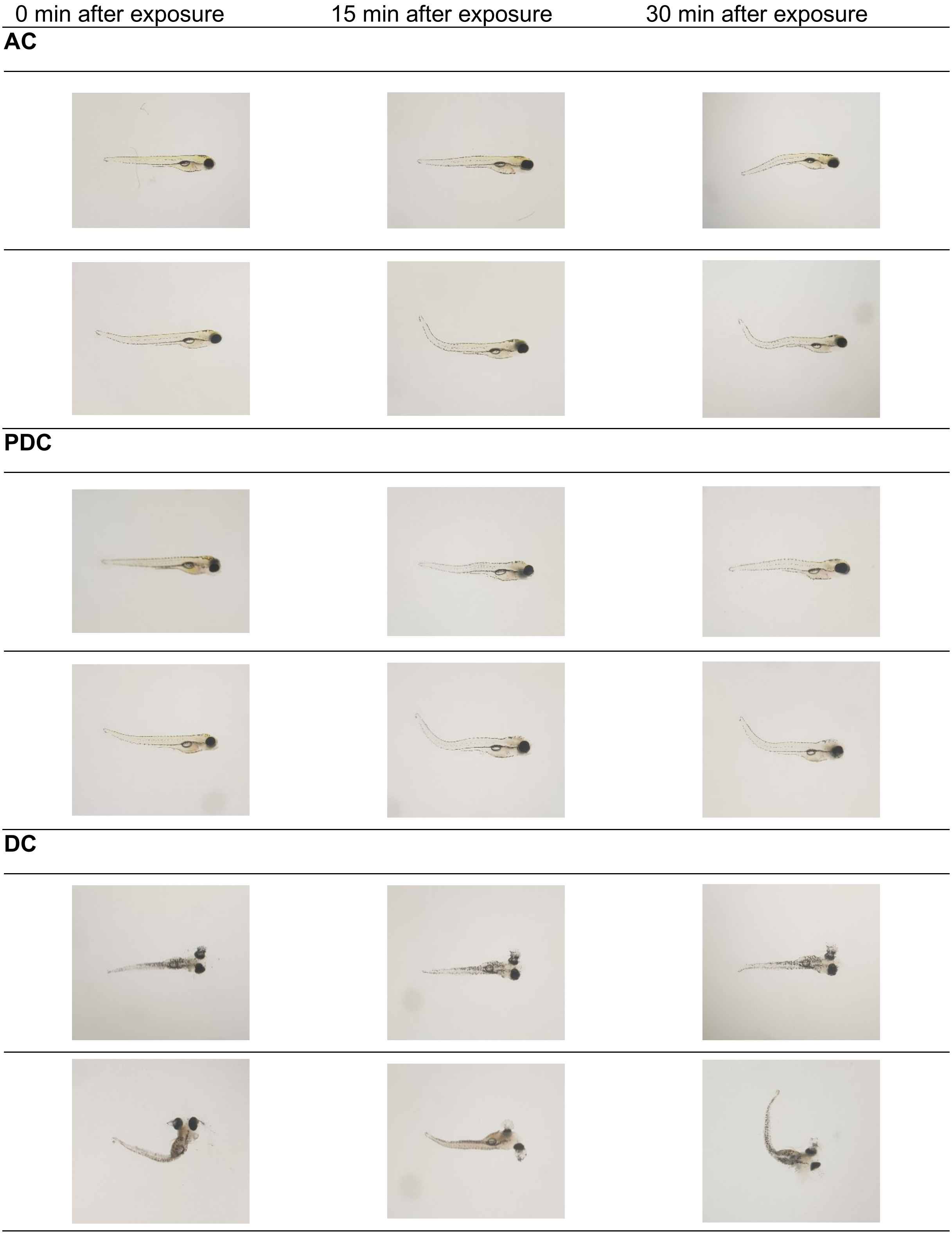

Example of morphological changes elicited by different voltage types at 50 V/cm, 32 s exposure duration. Each line shows the same fish at three time points.

Supplementary Figure 2:

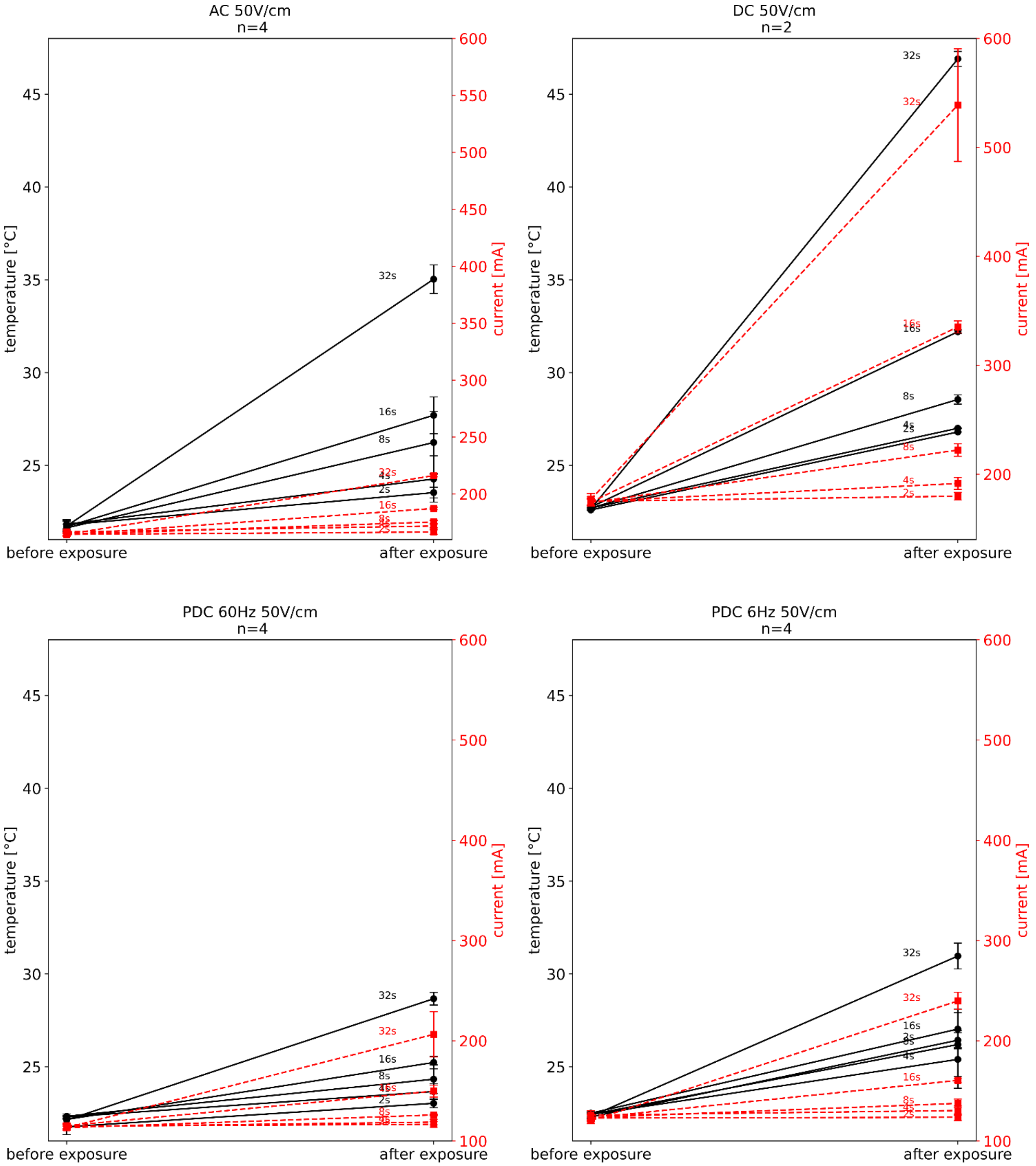

Temperature and current increase for all experiments conducted using the exposure chamber. Error bars indicate standard deviation across individual trials. N=4 trials for each data point.

Supplementary Figure 3:

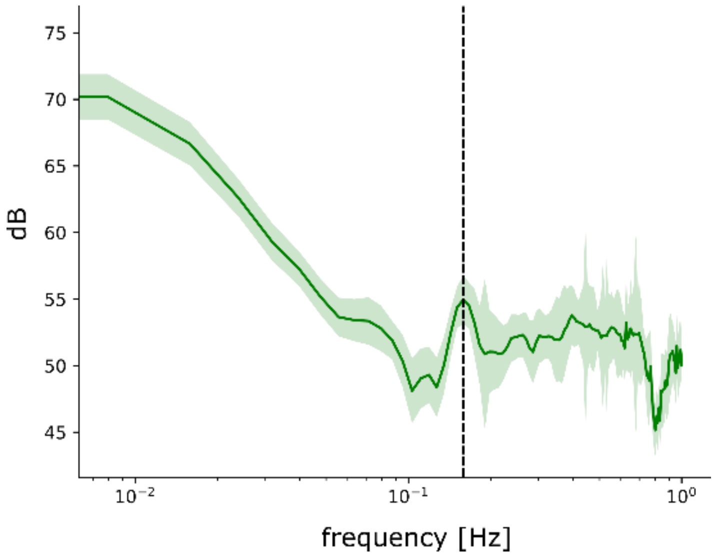

Power spectral density (PSD) analysis of baseline recordings with a 100-pixel drift over 720 frames. The visual stimulus main frequency at 0.1667 Hz is still clearly visible (dashed line). Envelope indicates the standard deviation.

Supplementary Figure 4:

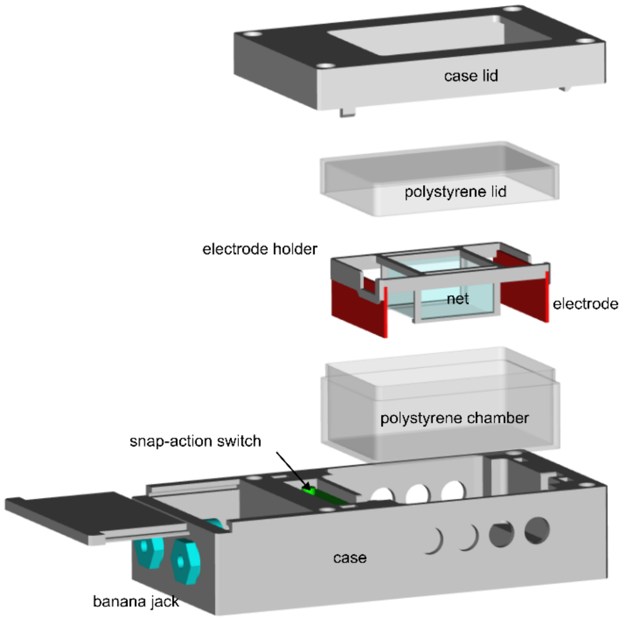

CAD drawing of the exposure chamber. The snap action switch was installed in the outer case. By closing the lid, the snap action switch is activated, and the circuit enabled. The exposure chamber was connected to the programmable power source via the banana jacks. The recess in the outer lid allowed video recording of the experiments.

Supplementary Figure 5:

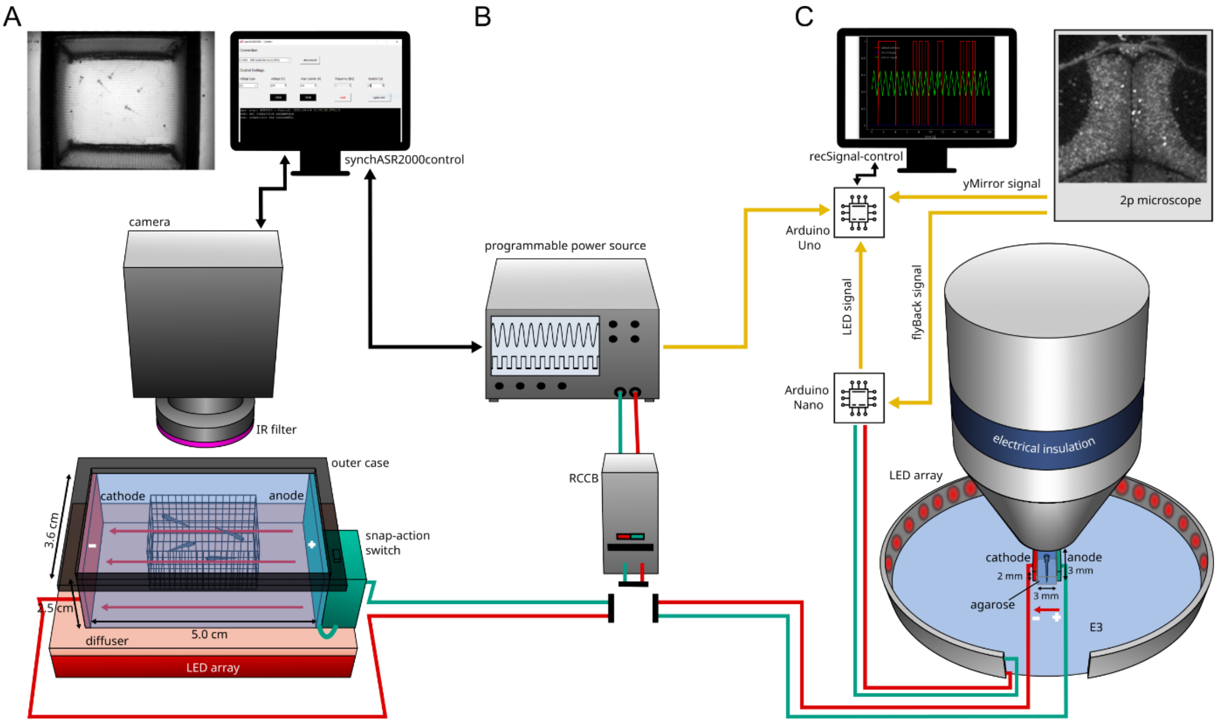

Experimental setups for free-swimming larvae and simultaneous electrical stunning and calcium imaging. **(A)** Setup for applying an electric field to multiple free-swimming larvae. Zebrafish larvae could swim between the electrodes within a rigid cuboid net, ensuring the positioning in the middle of the electric field. The lid of the outer case had to be closed to enable exposure. Electric fields as well as the IR-camera were controlled by the software synchASR2000-control. **(B)** Programmable power source with RCCB, generating electrical fields for either setup (A) or setup (B). **(C)** Setup for electrical stunning of single agarose-embedded larva and simultaneous calcium imaging alongside visual stimulation via an LED array. Data sampling was realized by using the custom software recSignal-control. The red arrow between the electrodes indicates the electrical field.

Supplementary Figure 6:

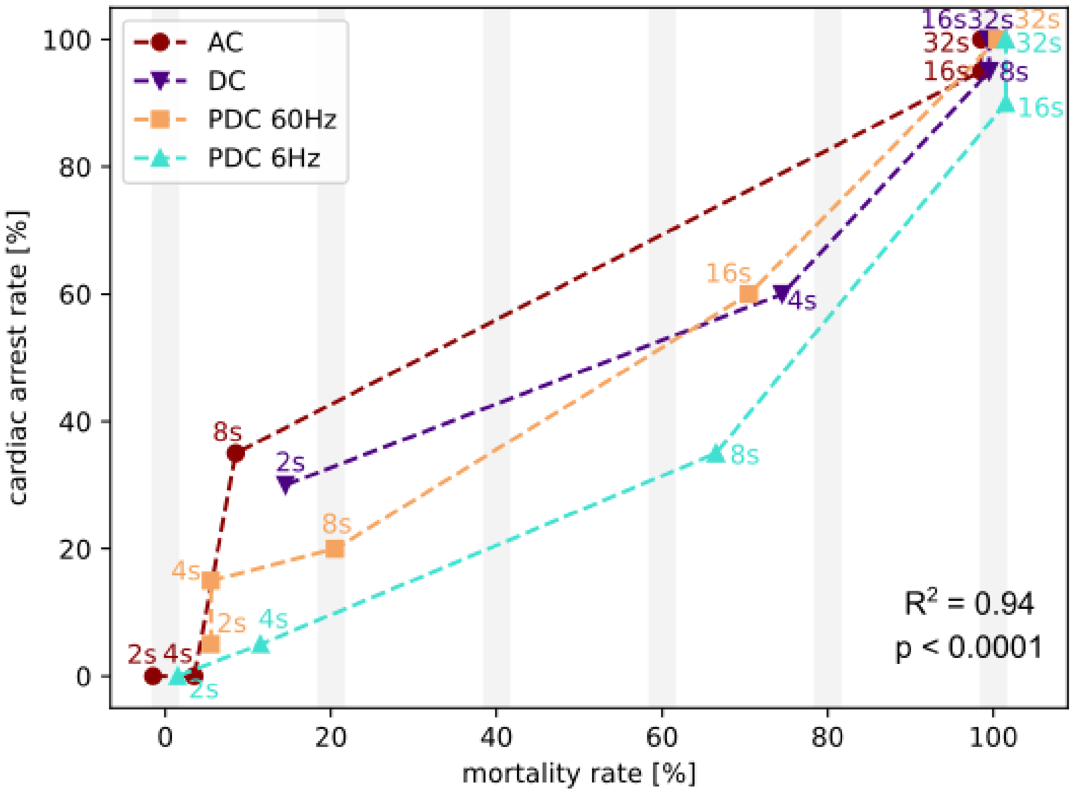

Relation between mortality rate and cardiac arrest for different voltage types and exposure durations at 50 V/cm.

Supplementary Figure 7:

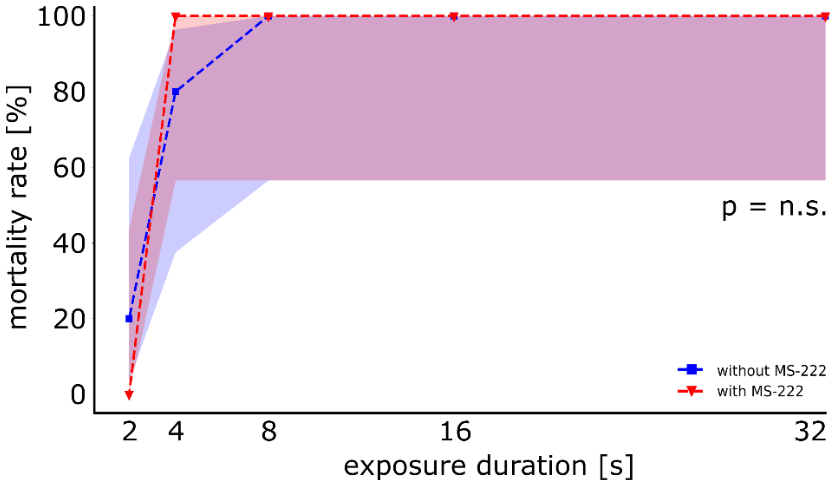

Mortality rates for DC voltage comparing MS-222 treated and untreated larvae. Envelopes show 95% Wilson confidence interval.

Supplementary Figure 8 :

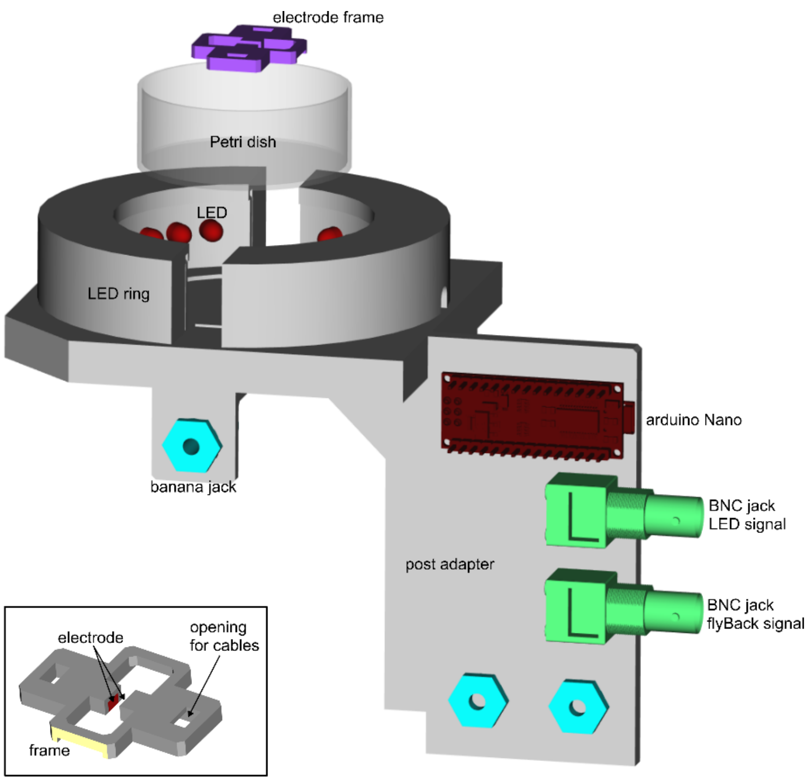

CAD drawing of the main setup components used for electrical stunning alongside visual stimulation and calcium imaging. LED ring, Petri dish with electrodes and the microcontroller (Arduino Nano) were compactly attached to a post. For easier handling, BNC jacks were attached for inputs and output cables. The setup was connected to the programmable power source via the banana jacks. Lower left corner: detailed view of the electrode frame with electrodes.

Supplementary Table 1:

	AC (60 Hz)						DC	PDC (60 Hz)	PDC (6 Hz)
	50 V/cm	*45 V/cm*	*40 V/cm*	*35 V/cm*	*30 V/cm*	*25 V/cm*	*50 V/cm*	*50 V/cm*	*50 V/cm*
**32 s**	20	20	20	20	20	20	20	20	20
**16 s**	20	20	20	20	20	20	20	20	20
**8 s**	20	X	X	X	X	X	20	20	20
**4 s**	20	X	X	X	X	X	20	20	20
**2 s**	20	X	X	X	X	X	20	20	20
	300						100	100	100

Total number of zebrafish larvae used in the mortality experiments. Each group / parameter combination (e.g. AC (60 Hz) + 50 V/cm + 32 s) was independently tested in four different trials with each trial containing 5 larvae (5 * 4 = 20). Those recordings were also used for determining the time to loss of equilibrium. X = not tested.

Supplementary Table 2:

	AC (60 Hz)						DC	PDC (60 Hz)	PDC (6 Hz)
	50 V/cm	*45 V/cm*	*40 V/cm*	*35 V/cm*	*30 V/cm*	*25 V/cm*	*50 V/cm*	*50 V/cm*	*50 V/cm*
**32 s**	7	X	X	X	X	X	X	X	X
**16 s**	X	X	X	X	X	X	X	X	X
**8 s**	X	X	X	X	X	X	X	X	X
**4 s**	X	X	X	X	X	X	X	X	X
**2 s**	X	X	X	X	x	X	X	X	X
	7						X	X	X

Total number of zebrafish larvae used in the calcium imaging experiments. Each group / parameter combination (e.g. AC (60 Hz) + 50 V/cm + 32 s) was independently tested in a single fish. X = not tested.

Supplementary Table 3:

	AC (60 Hz)						DC	PDC (60 Hz)	PDC (6 Hz)
	50 V/cm	*45 V/cm*	*40 V/cm*	*35 V/cm*	*30 V/cm*	*25 V/cm*	*50 V/cm*	*50 V/cm*	*50 V/cm*
**32 s**	2	X	X	X	X	X	X	X	X
**16 s**	X	X	X	X	X	X	X	X	X
**8 s**	X	X	X	X	X	X	X	X	X
**4 s**	X	X	X	X	X	X	X	X	X
**2 s**	X	X	X	X	x	X	X	X	X
	2						X	X	X

Total number of zebrafish larvae used in the high-speed recording tail tracking experiments. Each group / parameter combination (e.g. AC (60 Hz) + 50 V/cm + 32 s) was independently tested in a single fish. X = not tested.
